# Using Overt and Covert Items in Self-Report Personality Tests: Susceptibility to Faking and Identifiability of Possible Fakers

**DOI:** 10.3389/fpsyg.2018.01100

**Published:** 2018-07-03

**Authors:** Giulio Vidotto, Pasquale Anselmi, Luca Filipponi, Marco Tommasi, Aristide Saggino

**Affiliations:** ^1^Department of General Psychology, School of Psychology, University of Padova, Padova, Italy; ^2^Department of Philosophy, Sociology, Education and Applied Psychology, School of Psychology, University of Padova, Padova, Italy; ^3^Department of Developmental Psychology and Socialization, School of Psychology, University of Padova, Padova, Italy; ^4^Department of Psychological, Humanistic and Territorial Sciences, Università degli Studi “G. d’Annunzio” Chieti-Pescara, Chieti, Italy

**Keywords:** faking, overt, covert, psychological assessment, personality tests, Rasch models

## Abstract

Self-report personality tests widely used in clinical, medical, forensic, and organizational areas of psychological assessment are susceptible to faking. Several approaches have been developed to prevent or detect faking, which are based on the use of faking warnings, ipsative items, social desirability scales, and validity scales. The approach proposed in this work deals with the use of overt items (the construct is clear to test-takers) and covert items (the construct is obscure to test-takers). Covert items are expected to be more resistant to faking than overt items. Two hundred sixty-seven individuals were presented with an alexithymia scale. Two experimental conditions were considered. Respondents in the faking condition were asked to reproduce the profile of an alexithymic individual, whereas those in the sincere condition were not asked to exhibit a particular alexithymia profile. The items of the scale were categorized as overt or covert by expert psychotherapists and analyzed through Rasch models. Respondents in the faking condition were able to exhibit measures of alexithymia in the required direction. This occurred for both overt and covert items, but to a greater extent for overt items. Differently from overt items, covert items defined a latent variable whose meaning was shared between respondents in the sincere and faking condition, and resistant to deliberate distortion. Rasch fit statistics indicated unexpected responses more often for respondents in the faking condition than for those in the sincere condition and, in particular, for the responses to overt items by individuals in the faking condition. More than half of the respondents in the faking condition showed a drift rate (difference between the alexithymia levels estimated on the responses to overt and covert items) significantly larger than that observed in the respondents in the sincere condition.

## Introduction

Self-report personality tests, such as the Minnesota Multiphasic Personality Inventory-2 (MMPI-2; Butcher et al., 1989), the Eysenck Personality Questionnaire, (EPQ; Eysenck and Eysenck, 1975), the Millon Clinical Multiaxial Inventory-IV (MCMI-IV; Millon et al., 2015), and the Sixteen Personality Factor Questionnaire (16PF; Cattell et al., 1970), are widely used in clinical, medical, forensic, and organizational areas of psychological assessment (see, e.g., Domino and Domino, 2006; Rothstein and Goffin, 2006; Kaplan and Saccuzzo, 2009). An important limitation of these measures is that people can fake or distort responses. Faking occurs when respondents (a) engage in presentation behavior, framing a presentation of truth in a positive way; (b) lie; or (c) use only expediency as the criterion for making representations, without regard for either truth or falsehood (Levin and Zickar, 2002).

Several approaches have been developed to prevent or detect faking. Faking warning comprises a warning to test-takers that advanced approaches exist for detecting faking on the personality test that is being used. It may also include the information that adverse consequences will results for those who have been found to fake (Fluckinger et al., 2008). Literature supports faking warning as a viable approach to reducing, although not completely eliminating, faking (Goffin and Woods, 1995; Rothstein and Goffin, 2006). A meta-analysis by Dwight and Donovan (2003) indicated that faking warning may reduce faking by 30% on average, with larger reductions accompanying warnings that include mention of the consequences of faking detection. In addition, faking warning is inexpensive to add to an assessment program and can be easily combined with other approaches to faking reduction. However, there are some concerns associated with the use of this strategy for reducing faking. The validity of personality measures can be reduced by test-takers trying too hard to appear as though they are not faking (Dwight and Donovan, 2003). Faking warning has been found to increase the cognitive loading of personality trait scores (Vasilopoulos et al., 2005), that is the extent to which cognitive ability is assessed by the personality test. Cognitive loading may decrease the validity of personality measures because a given personality test score might be, to some extent, indicative of the test-taker’s level of cognitive ability as well as of his/her personality (Rothstein and Goffin, 2006).

Social desirability is the tendency of respondents to answer questions in a manner that will be viewed favorably by others, rather than how they truly feel or believe (King and Bruner, 2000). Elevate scores to social desirability scales have been taken as an indication of possible faking (van de Mortel, 2008), and “corrections” have been proposed that remove the effects of social desirability from personality test scores (Goffin and Christiansen, 2003; Sjöberg, 2015). However, there is evidence in the literature that social desirability is a poor indicator of faking (Zickar and Robie, 1999; Peterson et al., 2011), and that correcting personality test scores on the basis of social desirability does not improve the validity of measures (Christiansen et al., 1994; Ones et al., 1996; Ellingson et al., 1999).

The ipsative approach (or forced-choice approach) aims at obtaining more honest, self-descriptive responses to personality items by reducing the effect of perceived desirability of response options. This is achieved by presenting statements in pairs, triplets or quartets that have been equated with respect to perceived desirability (Rothstein and Goffin, 2006). The test-taker is instructed to choose the statement that best describes him/her. Because all the options have the same perceived desirability, there is no clear benefit to distort responses. Performance on one or more ipsative measures that falls below change to a statistically significant degree indicates biased responding. There is not clear evidence that tests with ipsative items reduce faking (Fluckinger et al., 2008), whereas they could increase the cognitive loading of trait scores, with a detrimental effect on the validity of measures (Christiansen et al., 2005). Moreover, test-taker reactions to these tests may be less positive than reactions to traditional tests (Harland, 2003).

The validity scales aim at measuring the extent to which respondents endorse items in a forthright manner. The validity scales of the Minnesota Multiphasic Personality Inventory (MMPI, Hathaway and McKinley, 1940, 1943), and those of its revisions, are among the most relevant examples. A type of validity scales are the lie scales, which aim at detecting attempts by respondents to present themselves in a favorable light. The logic beyond these scales is that only people who are high on social deception would endorse very improbable and trivial statements such as “I have never stolen anything, not even a hairpin.”. Professionals have been warned against the use of validity scales for detecting faking. If a person is highly motivated to present an average, yet different profile, he/she is likely to be able to accomplish that simulation without the validity scales detecting faking (Streicher, 1991). Respondents are able to reproduce without detection a specific profile (e.g., a creative artist), provided that they possess an accurate conception of the role to be simulated (Kroger and Turnbull, 1975).

The approach presented in this article takes into account whether the construct measured by the items is clear to test-takers or not. An item is called “overt” when the respondents immediately understand what the item is intended to measure. An item is called “covert” when the respondents (at least those without a thorough knowledge of the construct under investigation) are unaware of what the item measures. Covert items are expected to be more resistant to faking than overt items. Whenever test-takers have no idea about what the items are measuring, they cannot distort the responses in such a manner to present themselves in the desired way. Covert items have less face validity than overt items (Loewenthal, 2001). As a consequence, they demand a non-trivial knowledge of the construct to be correctly distorted in the desired direction.

The influence of faking on overt and covert items has been poorly investigated in the literature. Alliger et al. (1996) compared an overt and a covert integrity test in terms of their susceptibility to faking. The test scores of respondents who were asked to appear as honest as possible (faking condition) were compared with the test scores of respondents who were asked to answer the questions as candidly as possible (sincere condition). In the overt test, the respondents in the faking condition showed greater integrity than those in the sincere condition. No difference between the two conditions was found in the covert test.

The present study aims at investigating the influence of faking on overt and covert items, and the identifiability of possible fakers. The comparison between overt and covert is carried out at the level of the items, instead of being at the level of the different test (i.e., an overt test and a covert test). An overt test and a covert test measuring the same construct might differ with respect to the way in which the construct is defined. Conversely, the overt and covert items belonging to the same test derive from the same definition of the construct. Therefore, differences between the functioning of overt and covert items can be more easily attributed to the different clarity of the underlying construct, rather than to the different definition of the construct itself. Moreover, using one test instead of two reduces time and costs of the psychological assessment.

An analysis procedure is used, which is based on Rasch models (Rasch, 1960; Andrich, 1988; Bond and Fox, 2001). Rasch models characterize the responses of persons to items as a function of person and item measures, which, respectively, pertain to the level of a quantitative latent trait possessed by the persons or by the items. The specific meaning of these measures relies on the subject of the psychological assessment. In cognitive assessment, for instance, person measures denote the ability of persons, and item measures denote the difficulty of items. In this area, the higher the ability of a person relative to the difficulty of an item, the higher the probability that the person will give a correct response to the item. In health status assessment, person measures denote the health of persons, and item measures denote the severity of items. In this area, the higher the health of a person relative to the severity of an item, the higher the probability that the person will give to the item a response denoting absence of symptoms (e.g., a response “Not at all” to an item asking the person if he/she has trouble falling asleep). Applications of Rasch models for psychological assessment are well documented in the literature (see, e.g., Cole et al., 2004; Shea et al., 2009; Thomas, 2011; Anselmi et al., 2013, 2015; Da Dalt et al., 2013, 2015; Colledani et al., 2018; Sotgiu et al., 2018).

Several advantages derive from a Rasch analysis of faking. Rasch models allows for the transformation of non-linear, ordinal raw scores into linear, interval measures. Differently from ordinal scores, interval measures are characterized by measurement units that maintain the same size over the entire domain, so that measurement is more precise. Misusing ordinal raw scores as they were interval measures (e.g., calculating means and variances) is a common malpractice that can lead to erroneous conclusions (Merbitz et al., 1989; Kahler et al., 2008; Grimby et al., 2012). The measurement units constructed by Rasch models are called log-odds units or logits (Wright, 1993).

In the framework of Rasch models, the measures of respondents quantify the level of latent trait possessed by them. We expect the measures estimated on covert items to be less susceptible to faking than the measures estimated on overt items.

In addition to persons, Rasch models parameterize the items of the test. The location of the items on the latent trait defines the meaning of the variable which the items are intended to implement and, hence, its construct validity (Wright and Stone, 1999; Smith, 2001). Differently from overt items, we expect the covert items to implement a latent variable whose meaning is resistant to deliberate distortion. This means that the latent variables resulting by the responses of sincere respondents and fakers to covert items are expected to be similar, whereas the latent variables resulting by their responses to overt items are expected to be not.

In the framework of Rasch analysis, fit statistics are computed for each person and each item, that express the adherence between observed and expected responses. The fit statistics of a person quantify the extent to which his/her response behavior is consistent with that of the majority of people. These statistics might suggest, for instance, that the person has responded randomly or idiosyncratically, or that he/she has employed a particular response strategy (Smith, 2001; Linacre, 2009). Faking is a kind of response strategy (Frederiksen and Messick, 1959). We expect the fit statistics to reveal unexpected response behaviors more often for fakers than for sincere respondents. This is expected to occur more often for overt items, which should be more susceptible to faking.

## Materials and Methods

In the present work, a scenario was set up that concerns the faking of an alexithymia scale in personnel selection. Alexithymia is the inability to recognize, express and verbalize emotions. This construct was chosen because it is relatively little-known and, therefore, it is unlikely that individuals know how to distort their responses to covert items in the desired direction. Personnel selection was chosen because it is a high-stake setting in which individuals are highly motivated to fake. The occurrence of faking in personnel selection is well documented in the literature (see, e.g., Hough et al., 1990; Barrick and Mount, 1996; Ones et al., 1996; Hough, 1998; Rosse et al., 1998).

### Respondents

Two hundred sixty-seven university students, recruited from various degree courses at the University of Padova, took part in the study on a voluntary basis. Their mean age was 25.58 years (*SD* = 4.15), and 196 (73.41%) were female. All respondents gave written informed consent in accordance with the Declaration of Helsinki and anonymized for the analyses. The project has been approved, now as later, by the Ethical Committee for the Psychological Research of the University of Padova since a prospective ethics approval was not required at the time when the research was conducted (Protocol n. 2616).

### Measure of Alexithymia and Procedure

The Roman Alexithymic Scale (RAS; Baiocco et al., 2005) consists of 27 items, which are evaluated on a 4-point scale (Never-1, Sometimes-2, Often-3, and Always-4). Thirteen items are reverse. Greater scores indicate greater alexithymia.

The RAS was administered in individual sessions. All the respondents were asked to consider that they were applying for a job in which they were very interested. The respondents were randomly assigned to one of two conditions. The respondents in the faking condition were asked to reproduce the profile of an alexithymic individual. The instructions given to respondents in this condition were:

“Imagine you have responded to a job posting for a job that is prestigious, well-paid, and very important to you. The ideal candidate must be a person with a solid basic training and good skills in the use of computer programs. Good organizational skills, task-oriented objectives, emotional detachment, self-control, imperturbability, and no emotional involvement complete the profile. The received CVs will be selected on the basis of the requested requirements. Now, answer the questionnaire that I will present to you in such a way as to satisfy the conditions to be the ideal candidate.”

Conversely, the respondents in the sincere condition were not asked to exhibit a particular alexithymia profile. The instructions given to respondents in this condition were:

“Imagine you have responded to a job posting for a job that is prestigious, well-paid, and very important to you. The ideal candidate must be a person with a solid basic training and good skills in the use of computer programs. Good organizational skills and spontaneity complete the profile. The received CVs will be selected on the basis of the requested requirements. Now, answer the questionnaire that I will present to you in such a way as to satisfy the conditions to be the ideal candidate.”

### Categorization of the Items of the Roman Alexithymia Scale as “Overt” or “Covert”

Twenty-four expert psychotherapists were instructed about the meaning of “overt” and “covert” items, and were asked to categorize each of the 27 items of the RAS as overt or covert. The psychotherapists worked individually. Their evaluations were based on the content of the items and not on the response data.

For each item, **Table 1** presents the number of psychotherapists who categorized it as overt or covert. Twenty-one items were identified as overt (e.g., “I clearly recognize the emotions I feel”) and 6 as covert (e.g., “My physical sensations confuse me”). The agreement among psychotherapists was high for all the items. The lowest percentage of agreement was 87.50, and it was only observed for 2 items out of 27. There was perfect agreement (100%) for 17 items. The average agreement was 97.53%.

**Table 1:** Categorization of the items of the Roman Alexithymic Scale as “overt” or “covert”.

	Categorized as overt		Categorized as covert		Overall judgment
Item	*N*	%	*N*	%	
1	24	100	0	0	OVERT
2	0	0	24	100	COVERT
3	23	95.83	1	4.17	OVERT
4	22	91.67	2	8.33	OVERT
5	21	87.50	3	12.50	OVERT
6	24	100	0	0	OVERT
7	1	4.17	23	95.83	COVERT
8	24	100	0	0	OVERT
9	24	100	0	0	OVERT
10	23	95.83	1	4.17	OVERT
11	0	0	24	100	COVERT
12	23	95.83	1	4.17	OVERT
13	2	8.33	22	91.67	COVERT
14	24	100	0	0	OVERT
15	24	100	0	0	OVERT
16	21	87.50	3	12.50	OVERT
17	24	100	0	0	OVERT
18	23	95.83	1	4.17	OVERT
19	24	100	0	0	OVERT
20	24	100	0	0	OVERT
21	24	100	0	0	OVERT
22	1	4.17	23	95.83	COVERT
23	24	100	0	0	OVERT
24	0	0	24	100	COVERT
25	24	100	0	0	OVERT
26	24	100	0	0	OVERT
27	24	100	0	0	OVERT

Cohen’s *k* (Cohen, 1968) was computed on all the $\frac{24!}{2!(24-2)!}$ = 276 pairs of psychotherapists. The lowest agreement (*k* = 0.57) was observed in one pair only. Perfect agreement (*k* = 1) was observed in 68 pairs. The average agreement was *k̄* = 0.87 (*SD* = 0.10). Kendall’*W* (Kendall and Babington Smith, 1939) confirmed the high agreement among psychotherapists (*W* = 0.88, *df* = 26, *p* < 0.001).

### Data Analyses

Among the Rasch models, the rating scale model (RSM; Andrich, 1978) was chosen because the response scale of the RAS is polytomous and equal for all the items. The analyses were run using the computer program Facets 3.66.0 (Linacre, 2009). The responses to the reverse items were rescored prior to the analyses.

The functioning of the items and that of the response scale, as well as the internal consistency of the RAS were evaluated in all the analyses. The functioning of the items was evaluated through the infit and outfit mean-square statistics of the items. Their expected value is 1. Values greater than 2 (Wright and Linacre, 1994; Linacre, 2002b) for a specific item suggest that the item is badly formulated and confusing, or that it may measure a construct that is different from that measured by the other items (Smith, 2001; Linacre, 2009).

Likert scale structure requires that increasing levels of latent trait in a respondent correspond to increasing probabilities that he/she will choose higher response categories (Linacre, 2002a). The functioning of the response scale was assessed by determining whether the step calibrations (the points on the latent trait where two adjacent response categories are equally probable) were ordered or not (Linacre, 2002a; Tennant, 2004). If they were not ordered (i.e., if they did not increase monotonically while going up the response scale), then there would be discordance between the alexithymia level of respondents and the choice of the response categories. This would be interpreted as an indication that the response scale is not be adequate for measuring alexithymia.

The internal consistency of the RAS was evaluated through the separation reliability (*R*) of respondents (Fisher, 1992; Linacre, 2009). *R* is the Rasch equivalent of Cronbach’s α, but it is considered to be a better estimate of internal consistency for two main reasons (Wright and Stone, 1999; Smith, 2001). First, Cronbach’s α assumes that the level of measurement error is uniform across the entire range of test scores. Actually, the level of measurement error is generally larger for high and low scores than for scores in the middle of the range. This is due to the fact that, usually, there are more items designed to measure medium levels of the trait than items designed to measure extreme levels. In Rasch models, the estimate of each person measure has an associated standard error of measurement, thus differences in the level of measurement error among individuals are taken into account. Second, Cronbach’s α uses test scores for calculating the sample variance. Since test scores are not linear representations of the variable they are intended to indicate, the calculation of variance from them is always incorrect to some degree. Conversely, if the data fit the Rasch model, the measures estimated for each respondent are on a linear scale. Therefore, these measures are numerically suitable for calculating the sample variance.

Unidimensionality of the RAS was evaluated through infit and outfit mean-square statistics of the items, Wright’s unidimensionality index (WUI; Wright, 1994), and confirmatory factor analysis (CFA). Infit, outfit, and WUI are Rasch-based indicators of unidimensionality. Values of infit and/or outfit greater than 2 for a particular item suggest that the item may measure a construct that is different from that measured by the other items (Smith, 2001; Linacre, 2009). WUI is the ratio between the separation reliability of respondents based on asymptotic standard errors and the separation reliability of respondents based on misfit-inflated standard errors (Wright, 1994; Tennant and Pallant, 2006). Values above 0.9 are indicative of unidimensionality. CFA was run using Lisrel 8.71 (Jöreskorg and Sörbom, 2005). According to Schermelleh-Engel et al. (2003), fit is reasonable when χ^2^ is smaller than 3 × *df* (were *df* is the number of degrees of freedom), root mean square error of approximation (RMSEA) is smaller than 0.08, comparative fit index (CFI) is larger than 0.95, normed fit index (NFI) and goodness of fit index (GFI) are larger than 0.90.

#### Investigating the Influence of Faking on Overt and Covert Items

Three RSM analyses were run to investigate the influence of faking on overt and covert items. The first analysis was performed on the overall sample of respondents (*N* = 267). The responses to the overt items were considered separately from those to the covert items. This provided us with two measures for each respondent (parameters β), one denoting his/her alexithymia level estimated on the responses to overt items and the other denoting his/her alexithymia level estimated on the responses to covert items. It is worth noting that the estimates of parameters β are not influenced by the number of items. The estimates relative to overt and covert items were anchored to the same mean. Greater measures (i.e., larger logits) indicate higher alexithymia levels.

A 2 × 2 mixed factorial ANOVA was conducted, in which the condition (sincere, faking) was the between factor, and the item type (overt, covert) was the within factor. The dependent variables were the β estimates based on overt and covert items. We expect respondents in the faking condition to show greater alexithymia than respondents in the sincere condition. Since covert items are assumed to be less susceptible to manipulation than overt items, we expect the difference between the two conditions to decrease when the responses to covert items are considered.

The location of the items on the latent variable defines the meaning of the variable itself, thus providing information about construct validity (Wright and Stone, 1999; Smith, 2001). Two separate RSM analyses were conducted on respondents in the sincere and faking condition. These analyses provided us with two measures for each item (parameters δ), one estimated from the responses in the sincere condition and the other one estimated from the responses in the faking condition. Greater measures (i.e., larger logits) indicate items with fewer responses denoting alexithymia. The item measures concerning the two conditions were correlated. Since covert items should be more resistant to manipulation than overt items, we expect to found a stronger positive correlation between the measures of covert items than between the measures of overt items.

#### Investigating the Identifiability of Possible Fakers

This section presents two methods that could allow for identifying possible fakers. The first method is based on the infit and outfit mean-square statistics of the respondents. The expected value of both statistics is 1. Values greater than 2 (Wright and Linacre, 1994; Linacre, 2002b) for a specific respondent suggest that his/her response behavior is unexpected, given that exhibited by the majority of respondents. For example, he/she could have responded randomly or idiosyncratically, or he/she could have employed a particular response strategy (Smith, 2001; Linacre, 2009). Since faking is a kind of response strategy (Frederiksen and Messick, 1959), the fit statistics of respondents could allow the identification of possible fakers.

Five-hundred samples were generated, each one including all the 134 respondents in the sincere condition, plus 5 respondents randomly sampled from the faking condition. Therefore, the 500 samples differed from each other with respect to the 5 respondents sampled from the faking condition. Fit statistics allow the identification of respondents whose response behavior differs from that of the majority of respondents. For this reason, in each of the 500 samples, the number of possible fakers was kept small (5) compared to that of respondents who were asked to be sincere (134). The RSM was estimated on each sample, separately for the responses given to overt and covert items. We obtained, for each respondent of each sample, fit statistics based on the responses to overt items and fit statistics based on the responses to covert items. We expect the fit statistics to exceed the critical value of 2 more often for respondents in the faking condition than for respondents in the sincere condition. Overt items are assumed to be more susceptible to faking attempts than covert items. For respondents in the faking condition, we expect the fit statistics pertaining the responses to overt items to exceed 2 more often than those pertaining the responses to covert items. The *z* test was used for testing the statistical difference in the percentages of fit statistics greater than 2 between respondents of the two conditions, as well as between overt and covert items. Effect size of the *z* statistics was evaluated through odd ratio (*OR*). For each fit statistic (FS; infit or outfit) and each item type (overt or covert), an *OR* was computed as (*P*_faking FS > 2_ × *P*_sincere FS < 2_)/(*P*_faking FS < 2_ × *P*_sincere FS > 2_). For the respondents in the faking condition, an *OR* was computed for each fit statistic as (*P*_overt FS > 2_ × *P*_covert FS < 2_)/(*P*_overt FS < 2_ × *P*_covert FS > 2_).

The second method is based on computing a *drift rate* for each respondent, that is defined as the difference between his/her alexithymia level estimated on overt items and that estimated on covert items. For each respondent in the faking condition, it is tested if his/her drift rate is statistically larger than the average of the drift rates pertaining to the respondents in the sincere condition. The one sample *t*-test was used for this purpose. The rejection of the null hypothesis suggests that the respondent does not belong to the same population of the respondents in the sincere condition.

## Results

The Rasch-based statistics infit, outfit (smaller than 2 for all the items) and WUI (0.95), as well as the CFA-based statistics χ^2^ (739.39 < 3 × 281) and RMSEA (0.07) suggested that the RAS is substantially unidimensional. Conversely, the CFA-based statistics CFI (0.93), NFI (0.89), and GFI (0.72) suggested that there could be more than one dimension. These results do not allow for drawing certain conclusions about the unidimensionality of the RAS.

The step calibrations were ordered (the step calibrations Never-Sometimes, Sometimes-Often, Often-Always were −1.34, 0.52, 0.83 in the analysis on the overall sample; −1.93, 0.40, 1.53 in the analysis on respondents in the sincere condition; −0.99, 0.18, 0.81 in the analysis on respondents in the faking condition). This suggests that the response scale is adequate for measuring alexithymia.

The RAS has an adequate internal consistency (see **Table 2**). No relevant differences were found when the overall sample was considered, the respondents in the sincere condition only, or those in the faking condition only. The statistics *R* and α are affected by the number of items. The Spearman–Brown prophecy formula (Brown, 1910; Spearman, 1910) was used to predict the internal consistency of the covert items if their number was equal to that of the overt items (i.e., 21 items). Under this condition, the internal consistency of covert items largely resembled that of overt items.

**Table 2:** Internal consistency of the Roman Alexithymic Scale.

	Overall sample (*N* = 267)		Sincere condition (*N* = 134)		Faking condition (*N* = 133)	
Items	*R*	α	*R*	α	*R*	*α*
Entire scale (*N* = 27)	0.87	0.87	0.82	0.81	0.85	0.85
Overt items (*N* = 21)	0.86	0.87	0.82	0.81	0.84	0.84
Covert items (*N* = 6)	0.60	0.63	0.56	0.63	0.63	0.62
Covert items (*N* = 21) – predicted^a^	0.84	0.86	0.82	0.86	0.86	0.85

*R, Rasch separation reliability; α, Cronbach’s alpha. ^a^Predicted with the Spearman–Brown prophecy formula (Brown, 1910; Spearman, 1910).*

### Influence of Faking on Overt and Covert Items

**FIGURE 1:**
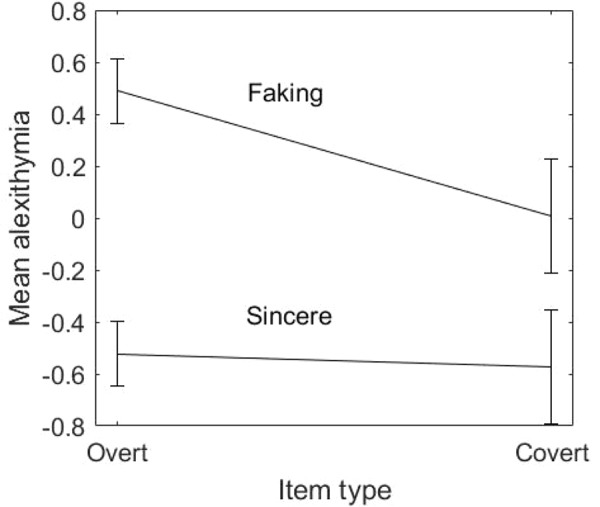
Average alexithymia (and 95% confidence intervals) of respondents in the sincere and faking condition, estimated on overt and covert items.

**Figure 1** depicts the average alexithymia level of respondents in the sincere and faking condition, estimated on overt and covert items separately. Respondents in the faking condition showed greater alexithymia than those in the sincere condition, both on the overt items [$\bar{\beta }$_faking–overt_ = 0.49, $\bar{\beta }$_sincere–overt_ = −0.52, *SE*_faking–overt_, *SE*_sincere–overt_ = 0.06, *t*(265) = 11.90, *p* < 0.001, Cohen’s *d* = 1.46] and on the covert items [$\bar{\beta }$_faking–covert_ = 0.01, $\bar{\beta }$_sincere–covert_ = −0.58, *SE*_faking–covert_, *SE*_sincere–covert_ = 0.11; *t*(265) = 3.79, *p* < 0.001, Cohen’s *d* = 0.47]. The interaction between condition and item type was significant, with the difference in alexithymia between respondents in the two conditions decreasing when responses to covert items were considered [*F*(1,265) = 7.65, *p* < 0.01, $\eta_{\text{p}}^{2}$ = 0.03]. Respondents in the faking group showed higher alexithymia on overt items than on covert items [*t*(132) = 3.83, *p* < 0.001, Cohen’s *d* = 0.40]. Respondents in the sincere group showed the same alexithymia on overt and covert items [*t*(133) = 0.46, *p* = 0.65, Cohen’s *d* = 0.04].

When the item measures estimated for the sincere condition and for the faking condition were correlated, a significant correlation was found between the measures of covert items (*r* = 0.92, *p* = 0.01) but not between those of overt items (*r* = 0.40, *p* = 0.07). The former correlation was significantly stronger than the latter (Fisher’s *z* = 1.87, *p* < 0.05). This result suggests that, differently from overt items, covert items define a latent variable whose meaning is shared between respondents in the sincere and faking condition, and resistant to deliberate distortion.

### Identifying Possible Fakers

About 5% of respondents in the sincere condition gave unexpected responses (infit/outfit > 2) to overt items (5.97% infit; 4.48% outfit) or covert items (5.22% infit; 4.48% outfit). In our study, this 5% can be taken as a benchmark for the percentage of respondents with unexpected response behavior that can be encountered among respondents who are expected to be sincere.

Across the 500 samples, about 35% of respondents in the faking condition gave unexpected responses to overt items (35.56% infit; 35.72% outfit), and about 19% to covert items (18.56% infit; 19.68% outfit). These percentages are greater than those observed in respondents in the sincere condition (*z* = 7.04, *OR* = 2.27 for infit on overt items; *z* = 7.43, *OR* = 11.85 for outfit on overt items; *z* = 3.93, *OR* = 4.14 for infit on covert items; *z* = 4.38, *OR* = 5.22 for outfit on covert items; *p* < 0.001 for all). For the respondents in the faking condition, unexpected responses were more frequent to overt items than to covert items (*z* = 13.53, *OR* = 2.42 for infit; *z* = 12.67, *OR* = 2.27 for outfit). These results suggest that both overt and covert items are susceptible to faking attempts, with overt items being to a greater extent.

The average drift rate of respondents in the sincere condition was 0.05 (*SD* = 1.32). Seventy-two respondents in the faking condition (out of 133; 54.14%) showed a drift rate significantly larger than 0.05 (Type-1 error probability = 0.05; Cohen’s *d* from 0.15 to 3.04), suggesting that they could belong to a population different from that of the respondents in the sincere condition.

## Discussion

The present study investigated the influence of faking on overt and covert items, and the identifiability of possible fakers. The investigations have been conducted on an alexithymia scale. The results were in line with expectations. Experimentally induced fakers were able to exhibit measures of alexithymia in the required direction. This occurred for both overt and covert items, but to a greater extent for overt items. Differently from overt items, covert items defined a latent variable whose meaning was shared between respondents in the sincere and faking condition, and resistant to deliberate distortion. Rasch fit statistics indicated unexpected responses more often for respondents in the faking condition than for those in the sincere condition and, in particular, for the responses to overt items by individuals in the faking condition. More than half of the respondents in the faking condition showed a drift rate (difference between the alexithymia levels estimated on the responses to overt and covert items) significantly larger than that observed in the respondents in the sincere condition.

We found that also covert items were susceptible to faking, although to a lesser extent than overt items. This is not in line with Alliger et al. (1996), who found no difference between the scores of the respondents who were asked to fake and those of the respondents who were asked to be sincere in a covert integrity test. Alliger et al. (1996) used an integrity test specifically developed as covert test. Differently, the items of the RAS were *a posteriori* categorized as overt and covert, instead of being specifically developed as overt or covert. The covert items of the RAS may be not as “covert” as items that are appositely though to be covert. The Rasch fit statistics indicated more unexpected responses to covert items by respondents asked to fake than by respondents asked to be sincere. This confirms the small, yet existing influence of faking on covert items, that has been found in the present study.

Two methods for identifying possible fakers have been proposed, which are based on the fit statistics of the respondents and on the computation of a drift rate. Results of the present study provide moderate evidence for the effectiveness of the two methods. It is worth noting that, once the Rasch model has been calibrated on unbiased data, it can be used for testing possible fakers without having to collect data on a new sample. Moreover, drift rate and fit statistics can be used for identifying possible fakers without having to add further tests (e.g., validity scales, social desirability scales) to the assessment program.

### Limitations and Suggestions for Future Research

Rasch models assume unidimensionality of the scale. A limitation of the present study is that unidimensionality of the RAS has not been supported with certainty. Multidimensionality, if present, could have influenced the estimation of person measures (Henning, 1988), with a detrimental effect on the functioning of the proposed approach. Future studies could investigate the functioning of the approach with scales whose unidimensionality is well-established.

Another limitation of the present study is that respondents in the faking condition were not asked about their perceived success in simulating the required profile. Future studies could investigate the relationship between the perceived success in simulating a profile and the responses to overt and covert items.

The items considered in the present study were *a posteriori* categorized as overt and covert, instead of being specifically developed as overt or covert. This could represent another limitation of the study, even if it is worth noting that psychotherapists agreed to a very large extend in categorizing the items. Future studies could investigate the functioning of items that are specifically developed as overt or covert.

A relatively little-known construct (alexithymia) was chosen to reduce the probability that individuals know how to distort their responses to covert items in the desired direction. Future studies should investigate the resistance of covert items to faking when the construct under evaluation is well-known.

A high-stake setting has been considered (personnel selection) in which individuals are highly motivated to fake. Future studies should investigate the functioning of overt and covert items in other areas of psychological assessment, such as clinical, medical, and forensic areas, which are affected by faking.

## Data Availability Statement

The raw data supporting the conclusions of this manuscript will be made available by the authors, without undue reservation, to any qualified researcher.

## Author Contributions

GV contributed conception and design of the study. LF conducted the research. PA and LF performed the statistical analyses. PA wrote the first draft of the manuscript. All authors contributed to manuscript revision, read and approved the submitted version.

## Conflict of Interest Statement

The authors declare that the research was conducted in the absence of any commercial or financial relationships that could be construed as a potential conflict of interest.
